# Primärmedizinische Versorgung in Deutschland am Scheideweg

**DOI:** 10.1007/s00108-024-01757-5

**Published:** 2024-08-09

**Authors:** Thomas Schröter

**Affiliations:** Kassenärztliche Vereinigung Thüringen, Zum Hospitalgraben 8, 99425 Weimar Deutschland, Deutschland

**Keywords:** Ärzte- und Schwesternmangel, Gesundheitskiosk, Primärversorgungszentren, Community Health Nurse, Hausarztambulanzen in Krankenhäusern, Physician and nurse shortage, Health kiosk, Primary care centers, Community Health Nurse, GP outpatient centers in hospitals

## Abstract

Über die bestehenden sozialgesetzlichen Optionen zur ärztlich verantworteten Primärversorgung gesetzlich Krankenversicherter hinaus gibt es mehrere Vorhaben zur Erweiterung der Angebotsstrukturen: Gesundheitskioske, Primärversorgungszentren und Community Health Nursing sowie hausärztliche Institutsambulanzen an Krankenhäusern. Diese neuen Projekte laufen auf eine Institutionalisierung bisher praxisgebundener Angebote und teilweise auf die Verlagerung bisher ärztlich verantworteter Heilkunde in die Eigenverantwortung nichtärztlicher Fachberufe hinaus – mit zusätzlichen finanziellen Aufwänden, aber ohne Schaffung neuer Versorgungskapazitäten. Die betrachteten Konstrukte erscheinen nicht geeignet, einen relevanten Beitrag zur Kompensation der Lücke bei Hausärzten und medizinischem Fachpersonal in einer demografisch bis etwa 2036 prognostizierten Kapazitätsschwundphase zu leisten. Die Weiterentwicklung des deutschen Sozialrechts zur Primärversorgung sollte stattdessen auf eine Effizienzsteigerung der knapper werdenden Ressourcen durch einen nach medizinischen Kriterien gesteuerten und gestuften Arztzugang fokussiert werden.

## Abstract (en)

In addition to the existing social law options for primary care under medical responsibility for those with statutory health insurance, there are several projects to supplement the service structures: healthcare kiosks, primary care centers and community health nursing as well as general practitioner outpatient clinics in hospitals. These new projects amount to an institutionalization of services that were previously based at outpatient offices and partially to the transfer of medical services that were previously the responsibility of doctors to the responsibility of non-physician healthcare professionals, with additional financial outlay but without creating new care capacities. The constructs considered do not appear to be suitable for making a relevant contribution to compensating for the gap in doctors specialized in general or internal medicine and in other medical professionals in a demographically induced capacity phasing out process that is forecast to last until around 2036. The further development of German social law on primary care should instead focus on increasing the efficiency of resources which are becoming scarcer through guided and graduated access to doctors according to medical criteria.

Die politischen Entscheidungsträger haben erkannt, dass die in Deutschland etablierte medizinische Versorgung mit den erwartbar sinkenden Personalressourcen so nicht aufrechtzuerhalten ist. Die aktuell verfolgten Ansätze zur Gegensteuerung konzentrieren sich auf die Primärversorgung. Sie treffen im Status quo auf ein flächendeckendes System inhabergeführter Hausarztpraxen, hausärztlicher Berufsausübungsgemeinschaften (BAG) sowie medizinischer Versorgungszentren (MVZ) mit Hausarztsitzen. Die Bedarfsplanung für hausärztliche Versorgungsaufträge zielt bei durchschnittlicher Altersstruktur auf einen Hausarztsitz pro 1607 Einwohner. Als selektivvertragliche Versorgungsangebote mit erhöhten Qualitätsanforderungen bzw. verbesserter Patientensteuerung werden Disease-Management-Programme (DMP) und die hausarztzentrierte Versorgung (HZV) angeboten. Damit stehen derzeit drei Wahloptionen für die Primärversorgung zur Verfügung (Tab. 1).

Derzeit stehen drei Wahloptionen für die Primärversorgung zur Verfügung

**Tab. 1:** Wahlentscheidung der Versicherten zur Primärversorgung im Status quo

*Unsystematische* anlassbezogene Inanspruchnahme irgendeines geeignet erscheinenden Vertragsarztes nach subjektivem Bedarf (§ 73 SGB V)
*Regelmäßige* Inanspruchnahme eines bestimmten Hausarztes, bei dem man sich zwecks Einhaltung wissenschaftlich empfohlener Behandlungspfade eingeschrieben hat, mit Hausarzt- und Facharztkonsultationen entsprechend den DMP-Vorgaben von Disease-Management-Programmen (§ 137f SGB V)
Grundsätzlich *primäre* Inanspruchnahme eines bestimmten Hausarztes, bei dem man sich für die hausarztzentrierte Versorgung eingeschrieben hat und der die weitere Behandlung anlassbezogen koordiniert (§ 73b SGB V)

*DMP* Disease-Management-Programm, *SGB* Sozialgesetzbuch

Im Koalitionsvertrag der Regierungskoalition und in den Initiativen zur Gesetzgebung bis Mitte 2024 fanden sich Ideen zu vier neuen Wahloptionen, die den Versicherten zukünftig angeboten werden könnten, um die demografisch bedingt prekär werdende hausärztliche Versorgung zu entlasten (Tab. 2). Dabei wird insbesondere die medizinische Versorgung chronisch Kranker im ländlichen Raum adressiert.

**Tab. 2:** Aktuelle Projekte zur zukünftigen Diversifizierung der Primärversorgung

Gesundheitskiosk
Community Health Nursing
Primärversorgungszentrum
Institutsermächtigung von Krankenhäusern mit sektorenübergreifendem Versorgungsauftrag für die hausärztliche Versorgung

Im vorliegenden Beitrag werden die bisher bekannten Vorstellungen zur Ausgestaltung der neuen Optionen kritisch beleuchtet und zielführendere Alternativen aufgezeigt.

## Fakten zum demografischen Handlungsbedarf

Die demografisch bedingte Zunahme des relativen Beanspruchungsindex von Hausarztpraxen in Deutschland bis 2035 wird in einer Untersuchung des Zentralinstituts für die kassenärztliche Versorgung (Zi) von 2023 mit 2 % im Bundesdurchschnitt angegeben [1]. Die Zahl von Ärzten, die in der hausärztlichen Versorgung tätig sind, zeigte nach 2003 einen steilen Rückgang, hat sich in den letzten 10 Jahren aber wieder positiv entwickelt [2]. Die Prognosen gehen dennoch von einer dramatischen Ausdünnung in den Bedarfsplanungsbereichen für die hausärztliche Versorgung in den nächsten 15 Jahren aus. In einer Studie des IGES Instituts im Auftrag der Robert Bosch Stiftung von 2021 wird das Fehlen von etwa 11.000 Hausärzten im Jahr 2035 hergeleitet [3]. Die aktuelle Zi-Hochrechnung des nichtgedeckten Bedarfs an Absolventen des Medizinstudiums als Nachwuchs für die ambulante und stationäre Versorgung zeigt, dass das Nachbesetzungsdefizit noch bis etwa 2036 anhält. Danach werde der Tiefpunkt von etwa 74 % der vertragsärztlichen Behandlungsleistung in Relation zum Niveau von 2021 voraussichtlich nicht weiter unterschritten [4].

Von einer analogen Dynamik des Fachkräftemangels beim nichtärztlichen medizinischen Fachpersonal ist auszugehen. Laut Analyse der Bundesagentur für Arbeit gehören die medizinischen Fachberufe schon jetzt zu den besonders von Engpässen betroffenen Gruppen [5]. Dies belegte auch die Sonderbefragung im Rahmen des Zi-Praxis-Panels (ZiPP) von 2021 bezüglich der medizinischen Fachangestellten in Arztpraxen [6, 7]. Aus der Alterspyramide der Gesamtbevölkerung [8] kann im Zusammenhang mit den vorstehend zitierten Problemanalysen darauf geschlossen werden, dass die anstehende Ruhestandswelle und fehlender Nachwuchs beim Praxispersonal in der Primärversorgung kein geringeres Problem darstellen als in der Ärzteschaft. Dies bestätigte auch das aktuelle Gutachten des Sachverständigenrats für Gesundheit & Pflege [9].

## Gesundheitskioske

Der Begriff bezeichnet eine politische Idee, die von der SPD in den Koalitionsvertrag der amtierenden Bundesregierung [10] eingebracht und in einem Vorentwurf zum Gesundheitsversorgungsstärkungsgesetz (GVSG; [11]) näher ausgeführt wurde. Im Hinblick auf die medizinischen Leistungsanteile in Gesundheitskiosken wird auf die *Beratung* zu kurativen und präventiven medizinischen Leistungen sowie auf die *Vermittlung* zu entsprechenden Leistungsanbietern fokussiert. Bürgerinnen und Bürger in strukturell benachteiligten Stadtteilen und Regionen sollen darüber hinaus *unmittelbare Unterstützung bei der Klärung gesundheitlicher und damit verbundener sozialer Angelegenheiten* bekommen. Auch die *Übernahme einfacher medizinischer Routineaufgaben* im Rahmen ärztlicher Delegation durch den Gesundheitskiosk ist vorgesehen. Zudem soll hier eine *Koordinierung* verschiedener Gesundheitsleistungen einschließlich ambulanter telemedizinischer Angebote erfolgen. Damit handelt es sich eindeutig um eine Überlappung mit den hausärztlichen Versorgungsformen in Tab. 1, die derzeit auch schon die Koordination diagnostischer, therapeutischer und pflegerischer Maßnahmen, die Integration nichtärztlicher Hilfen, die Kooperation mit flankierenden Diensten sowie die Versorgungssteuerung beinhalten. Die Leitung soll eine Pflegefachkraft übernehmen, die Zusammenarbeit mit dem öffentlichen Gesundheitsdienst obligat sein [11].

Parallelstrukturen zur hausärztlichen Versorgung erhöhen nicht die Effizienz

Neben der Wohltätigkeitsfunktion einer kommunalen „Kümmerstelle“ für Menschen mit Unterstützungsbedarf handelt es sich beim Gesundheitskiosk um eine substanzielle Parallelstruktur zur hausärztlichen Versorgung, beide sind in gleicher Weise niedrigschwellig zugänglich. Das Konstrukt bewirkt eine Unschärfe in den Zuständigkeiten zweier unabhängig nebeneinander bestehender Angebote (Doppelvorhaltung). Die Schaffung von Gesundheitskiosken würde Arbeitskräfte benötigen, die in der bestehenden hausärztlichen Versorgung jetzt schon zunehmend fehlen und nun perspektivisch vermehrt entzogen werden. Dass klassische Aufgaben, die in hausärztlichen Teams seit jeher auf Gesundheitsfachberufe delegiert werden, zusätzlich auch in Gesundheitskiosken angeboten werden sollen, erzeugt keine Effizienzerhöhung der knappen Personalressourcen. Im Referentenentwurf des Bundesgesundheitsministeriums zum GVSG [12] und im Regierungsentwurf [13] sind Gesundheitskioske nicht mehr enthalten. Es ist aber zu erwarten, dass die Regelungsabsicht im parlamentarischen Verfahren weiterverfolgt wird.

## Community Health Nursing

Das 30 Jahre lang in der ehemaligen DDR bestehende Berufsbild der Gemeindeschwester zeigt Parallelen zum Konzept der Community Health Nurse (CHN), wie es derzeit von der Robert Bosch Stiftung und vom Deutschen Berufsverband für Pflegeberufe in Deutschland sowie von dessen Agnes-Karll-Gesellschaft propagiert wird. Es finden sich aber auch deutliche Unterschiede. Die wesentlichsten Unterschiede bestehen im akademischen Upgrading auf einen Master of Arts als Qualifikationsniveau und im Anspruch auf die selbstständige Ausübung der Heilkunde [14]. Das neue Berufsbild ist seit 2015 als nichtärztliche Leitungsqualifikation für patientenorientierte Zentren zur Primär- und Langzeitversorgung (PORT-Zentren) entwickelt worden. Es wird derzeit vom Bosch Health Campus in Stuttgart als vermeintliche Lösung des Ärztemangels propagiert [15].

Im Nachbarland Österreich werden seit 2022 CHN mit Diplomabschluss in Pilotprojekten eingesetzt, die von Kommunen oder Sozialhilfeverbänden beantragt werden können. Laut Weltgesundheitsorganisation (WHO) handelt es sich um eine Querschnittsdisziplin, „welche Fähigkeiten aus der Gesundheits- und Krankenpflege mit jenen von Public Health und dem Sozialbereich verbindet“. International gibt es keine einheitliche Bezeichnung, kein übereinstimmend definiertes Berufsbild, unterschiedliche Qualifikationsniveaus und uneinheitliche Verknüpfungen mit anderen Gesundheitsdienstleistern. In Finnland, Norwegen, Schweden, Großbritannien, Irland und Kanada arbeiten schon länger etablierte CHN in voneinander abweichenden Settings, mit unterschiedlichen Zielgruppen und Schwerpunktaufgaben. Übereinstimmungsmerkmale sind die Hausbesuchstätigkeit und die gemeindeorientierte Versorgung [16].

Von der Schaffung eines neuen Heilkundeberufs neben dem Arztberuf, der sich im akademischen Zeitaufwand nicht wesentlich von der ärztlichen Ausbildung unterscheidet, ist unter demografischen Gesichtspunkten kein relevanter Beitrag zur Problemlösung zu erwarten. Die irreführende Übernahme der Berufsbezeichnung „Community Health Nurse“ von den im Bachelor-Niveau angesiedelten Gemeindeschwestern anderer westlicher Länder durch die Robert Bosch Stiftung begünstigt die Verschleierung der Zielsetzung des Projekts: Statt einer sinnvollen Delegation primärmedizinischer Leistungen soll hier die weitgehende Substitution hausärztlicher Tätigkeit durch eine nichtärztliche Qualifikation verwirklicht werden.

## Primärversorgungszentren

Das vorstehend erwähnte PORT-Zentrum des Bosch Health Campus in Stuttgart verfolgt einen völlig anderen Ansatz als die Primärversorgungszentren nach den Vorstellungen des Hausärztinnen- und Hausärzteverbands, der ebenfalls probeweise in Baden-Württemberg das Konzept „Hausärztliches Primärversorgungszentrum – Patientenversorgung Interprofessionell“ (HÄPPI; [17]) umsetzen will. Bei HÄPPI steht die hausärztlich geführte Teampraxis mit Leistungsdelegation an akademisierte Pflegeberufe im Vordergrund.

Im bereits erwähnten Vorentwurf zum GVSG wurde unter dem Terminus Primärversorgungszentrum (PVZ) eine neue Angebotsform hausärztlicher Versorgung definiert. Die PVZ sollen sich von herkömmlichen Hausarztpraxen bzw. BAG und MVZ mit Hausärzten durch „zusätzliche berufsgruppenübergreifende, koordinierte, kooperative und versorgungssteuernde Versorgungselemente“ unterscheiden. Verpflichtend sind dabei nur die Beschäftigung mindestens einer besonders qualifizierten Pflegefachperson und der Abschluss einer Kooperationsvereinbarung mit kommunalen Stellen [11].

Primärversorgungszentren nach GVSG-Vorentwurf haben keinen innovativen Mehrwert für die Versorgung

In der Realität könnten alle Merkmale der im GVSG-Vorentwurf beschriebenen PVZ allerdings bereits heute – völlig unreguliert – von hausärztlichen Praxisgemeinschaften, BAG und MVZ angeboten werden, ohne sich so zu nennen. PVZ hätten somit keinen innovativen Mehrwert für die Versorgung. Bemerkenswert bleibt, welche unterschiedlichen Konzepte verschiedene Stakeholder (unter anderem der Bundesverband Managed Care [18]) unter dem gleichen Schlagwort verfolgen. Das Konstrukt ist in späteren Versionen des Gesetzentwurfs [12, 13] nicht mehr enthalten, wird aber absehbar über den Bundesrat wieder in die parlamentarische Debatte gelangen.

## Hausärztliche Institutsambulanzen an Krankenhäusern

Mit dem Entwurf zum Krankenhausversorgungsverbesserungsgesetz (KHVVG; [19]) ist ein politischer Vorstoß unternommen worden, der den Anspruch von Krankenhäusern mit sektorenübergreifendem Versorgungsauftrag auf die Ermächtigung zur hausärztlichen Versorgung zum Inhalt hat – sofern der Krankenhausstandort nicht in einem Bereich mit Zulassungsbeschränkungen liegt. Ein sektorenübergreifender Versorgungsauftrag wird von den Planungsbehörden voraussichtlich jedem Krankenhaus zugesprochen werden, das ihn begehrt – 80 % der hausärztlichen Planungsbereiche sind nicht gesperrt. Damit kann die hausärztliche Ambulanz an den meisten Krankenhäusern zum Regelfall werden.

Die Möglichkeit, über hausärztliche Institutsambulanzen mit großzügiger Indikationsstellung Betten der sektorenübergreifenden Versorgung zu füllen und ohne Prüfmöglichkeit durch den Medizinischen Dienst tagesgleiche Pflegesätze zu erlösen, lockt als attraktives Geschäftsfeld zur Rettung bisher unwirtschaftlicher Kliniken. Für Transformationskosten sollen zudem jährlich 5 Mrd. € im Bund zur Verfügung stehen [19]. Allerdings müssten für die neuen Ambulanzen Hausärzte angestellt werden, die man nur aus dem vertragsärztlichen Bestand akquirieren kann. Institutsambulanzen lassen sich somit zwanglos in die Reihe der Konstrukte ohne zusätzliches Versorgungspotenzial einordnen. Aber sie haben – ebenso wie Gesundheitskioske und CHN – das Potenzial zu disruptiven Veränderungen der Strukturen des deutschen Gesundheitswesens. Allein die veränderte Allokation der demografisch schwindenden ärztlichen und nichtärztlichen Personalressourcen hebt an keiner Stelle Effizienzreserven. Das Vorhaben einer Institutionalisierung der hausärztlichen Versorgung in Krankenhäusern ist aber geeignet, die inhabergeführte Praxis als tragende Säule der ambulanten Versorgung schleichend abzuwickeln.

## Alternativen

Angesichts der Tatsache, dass die Zahl verfügbarer Ärzte sowie die Zahl des medizinischen Fachpersonals bei leicht steigendem Versorgungsbedarf der Bevölkerung in den nächsten 12 Jahren unaufhaltsam zurückgeht, erschiene es sinnvoller, die ordnungspolitische Weichenstellung jetzt auf eine Effizienzerhöhung der Bestandsstrukturen auszurichten. Dieses Ziel wäre auf zwei Wegen erreichbar:

- Durch hierarchische Zugangssteuerung in einem gestuften Primärversorgungssystem
- Durch Sanktionierung ungesteuerter Inanspruchnahmen

Beide Maßnahmen bedeuten Einschränkungen der Möglichkeit für gesetzlich Krankenversicherte, bei jeglichen Befindlichkeitsstörungen unbegrenzt ärztliche Leistungen im Sachleistungsprinzip in Anspruch nehmen zu können. Politische Mehrheiten für die gesetzgeberische Abschaffung dieses deutschen Luxus werden realistischer, je stärker ein manifester Versorgungsnotstand für die Wähler spürbar wird. Die nachstehenden Ansatzpunkte zu einer Lösung könnten im Rahmen eines freiwilligen beitragsbegünstigten Tarifs der gesetzlichen Krankenversicherung solchen dramatischen Entwicklungen zuvorkommen.

Die zunehmende Digitalisierung des Gesundheitswesens schafft das Potenzial, dass Personen mit gesundheitlichen Problemen oder Fragen zunächst eine *telemedizinische Primärberatung* in Anspruch nehmen, bevor sie Versorgungseinrichtungen aufsuchen. Die bundesweit verfügbaren Medizinprodukte zur strukturierten medizinischen Ersteinschätzung im Bereitschaftsdienst der Kassenärztlichen Vereinigungen [20] sind dafür eine Blaupause. Eine wirksame Entlastung der Primärversorgung entsteht erst dadurch, dass medizinisch valide Steuerungsinstrumente vor jedem Arztkontakt für Patienten obligat werden.

Als Träger für eine allgemeine telemedizinische Primärberatung bieten sich *Teampraxen zur interprofessionell-arbeitsteiligen Patientenversorgung unter ärztlicher Leitung* an, zu denen sich die herkömmlichen Hausarztpraxen derzeit entwickeln. Die Profilierung von medizinischen Fachangestellten zu den als nichtärztliche Praxisassistenz (NÄPA), Versorgungsassistentin/-assistent in der Hausarztpraxis (VERAH) oder agnes^zwei^ bezeichneten Hausbesuchsassistentinnen und -assistenten und die Ausbildung von Physician Assistents (Bachelor of Arts) sind als adäquate Antwort der Beteiligten auf die demografischen Herausforderungen seit mehreren Jahren im Gange. Die Beratung durch solche qualifizierten Mitarbeiterinnen und Mitarbeiter könnte regelhaft dem Arztkontakt vorgeschaltet werden und einen Teil der Praxiskonsultationen ohne Arztkontakt abschließen. Vergütungen für den – gegebenenfalls auch telemedizinischen – Teamkontakt und für das Case-Management sollten nicht an einen obligaten Arztkontakt der Patienten geknüpft werden. Hierzu sind gesetzgeberische Aufträge an die Selbstverwaltung ausreichend; die Verlagerung medizinischer Leistungen in neu zu schaffende Anlaufstellen (Tab. 2) würde sich erübrigen.

Einer grundlegenden gesetzlichen Regeländerung bedürfte es darüber hinaus, um die *obligate primärmedizinische Versorgung durch hausarztgeführte Teampraxen als Tarifoption in der kollektivvertragsärztlichen Regelversorgung* zu verankern. Mit einem solchen Schritt könnte das Massenphänomen der Fehlinanspruchnahme weiterer ärztlicher Kapazitäten zurückgedrängt werden. Versicherte in diesem Tarif verzichten freiwillig auf den ungesteuerten Direktzugang zum Facharzt und lassen sich vor dem Aufsuchen von Notfallzentren telemedizinisch ersteinschätzen. Das Gatekeeping zur fachärztlichen Mitbehandlung könnte bei zahlreichen Anlässen, beispielsweise bei Patienten mit chronischer Erkrankung, an qualifiziertes Fachpersonal delegiert werden, um eine Überlastung der Hausärzte durch Routinen im „Primärpraxistarif“ zu vermeiden. Eine solche Tariflösung wäre effektiver als die HZV nach § 73b Sozialgesetzbuch (SGB) V, in der es ohne Konsequenzen bleibt, wenn Patienten entgegen ihrer vertraglichen Verpflichtung Ärztehopping betreiben. Die Evaluation in Baden-Württemberg hat gezeigt, in welch hohem Maß die ungesteuerte Inanspruchnahme auch in der HZV das ambulante Versorgungssystem belastet^1^. Dieser Ansatz ist zudem nicht zielführend, weil er selektiv begünstigt, statt kollektivvertraglich zu steuern. Das demografisch belastete System benötigt eine wirksame Drosselung der ungesteuerten Inanspruchnahme.

## Fazit

- Keines der derzeit regierungsseitig erwogenen Projekte löst die demografischen Probleme in der medizinischen Primärversorgung.
- Ärztliche Organisationen sollten offensiv für einen alternativen Lösungsansatz eintreten, der dem demografisch bedingt zunehmenden Ärzte- und Fachpersonalmangel in den Jahren bis 2036 ohne kostenintensive und potenziell disruptive Systemveränderungen begegnet.
- Die Einführung eines Wahltarifs der gesetzlichen Krankenversicherung mit Anreizen zur Patientensteuerung als primärpraxiszentrierte Versorgung im Kollektivvertragssystem wäre für die ambulante Grundversorgung der Versicherten effektiver als Gesundheitskioske, Community Health Nurses oder hausärztliche Versorgung an Krankenhäusern.
- Zur Ausweitung von Telemedizin und nichtärztlichem Case-Management ist keine Revolution der Strukturen erforderlich.

## Footnotes

1 Szecsenyi J et al., Gerlach FM et al.: Evaluation der Hausarztzentrierten Versorgung (HZV) nach § 73b SGB V in Baden-Württemberg (2011 bis 2018), Studienphase 2019 bis 2020. Teil 1 Universitätsklinikum Heidelberg, Abteilung Allgemeinmedizin und Versorgungsforschung 2021. Teil 2 Johann Wolfgang Goethe-Universität Frankfurt am Main, Institut für Allgemeinmedizin 2021. Nicht veröffentlicht
